# Microalgae-Enabled Wastewater Remediation and Nutrient Recovery through Membrane Photobioreactors: Recent Achievements and Future Perspective

**DOI:** 10.3390/membranes12111094

**Published:** 2022-11-03

**Authors:** Pei Sean Goh, Nor Akalili Ahmad, Jun Wei Lim, Yong Yeow Liang, Hooi Siang Kang, Ahmad Fauzi Ismail, Gangasalam Arthanareeswaran

**Affiliations:** 1 Advanced Membrane Technology Research Centre (AMTEC), Faculty of Chemical and Energy Engineering, Universiti Teknologi Malaysia, Skudai 81310, Johor, Malaysia; 2 HICoE-Centre for Biofuel and Biochemical Research, Institute of Self-Sustainable Building, Department of Fundamental and Applied Sciences, Universiti Teknologi PETRONAS, Seri Iskandar 32610, Perak Darul Ridzuan, Malaysia; 3 Department of Biotechnology, Saveetha School of Engineering, Saveetha Institute of Medical and Technical Sciences, Chennai 602105, India; 4 Faculty of Chemical and Process Engineering Technology, Universiti Malaysia Pahang, Lebuhraya Tun Razak, Gambang, Kuantan 26300, Pahang, Malaysia; 5 Marine Technology Centre, Institute for Vehicle System & Engineering, Faculty of Mechanical Engineering, Universiti Teknologi Malaysia, Skudai 81310, Johor, Malaysia; 6 Membrane Research Laboratory, Department of Chemical Engineering, National Institute of Technology, Tiruchirappalli 620015, India

**Keywords:** microalgae, membrane photobioreactor, wastewater treatment, nutrient recovery, bioremediation

## Abstract

The use of microalgae for wastewater remediation and nutrient recovery answers the call for a circular bioeconomy, which involves waste resource utilization and ecosystem protection. The integration of microalgae cultivation and wastewater treatment has been proposed as a promising strategy to tackle the issues of water and energy source depletions. Specifically, microalgae-enabled wastewater treatment offers an opportunity to simultaneously implement wastewater remediation and valuable biomass production. As a versatile technology, membrane-based processes have been increasingly explored for the integration of microalgae-based wastewater remediation. This review provides a literature survey and discussion of recent progressions and achievements made in the development of membrane photobioreactors (MPBRs) for wastewater treatment and nutrient recovery. The opportunities of using microalgae-based wastewater treatment as an interesting option to manage effluents that contain high levels of nutrients are explored. The innovations made in the design of membrane photobioreactors and their performances are evaluated. The achievements pave a way for the effective and practical implementation of membrane technology in large-scale microalgae-enabled wastewater remediation and nutrient recovery processes.

## 1. Introduction

With the exponential growth of the human population along with urbanization, industrialization, and agricultural activities, maintaining fresh water supplies to meet the increasing demands has become a difficult proposition [1]. The distressing circumstances have awakened the interest in considering the possibility of reclaiming existing wastewater for reuse [2,3]. On the other hand, the concerns regarding freshwater resource and environmental pollution have constantly urged for the upgrading of existing wastewater treatment processes. In an era where a waste-derived economy is highlighted as the method to attain environmental sustainability, the establishment of efficient technologies that cater to both wastewater treatment and useful resource recovery is in high demand [4,5,6]. The removal of contaminants such as dissolved and suspended substances from municipal wastewater or industry wastewater can be performed through various technologies, which can be further classified into physical, chemical, and biological means [7,8]. Regardless of the mechanisms involved, the primary goal of these wastewater treatment technologies is to remove pollutants such as organic matters, heavy metals, pharmaceutically active products, and nutrients in order to achieve the standards that fit the intended application of the treated wastewater. The hybridization of these technologies has become more prevailing and has been actively studied owing to its effectiveness in handling a wide range of contaminants present in different wastewater sources [9,10,11].

Microalgae, which have been referred to as a ‘green gold’ conferred by nature, can be widely applied as environmentally sustainable resources. The use of microalgae as an alternative energy source has been explored since more than five decades ago, when algae-based biodiesel started to take off [12]. Besides serving as sustainable feedstocks for the production of biofuels [13], microalgae have been crowned as a versatile tool for bioremediation due to their potential for adsorbing carbon dioxide through photosynthesis. This mechanism can be used as an alternative method to reduce the emission of greenhouse gas from various anthropogenic sources and activities, including power plants. As a consolidated solution to tackle environmental challenges, the harvested algae biomass can serve as a resource to produce biogas and biofuel along with many other types of energy carriers. More recently, microalgae have also been exploited for wastewater treatment [14]. Compared with the typically used physical, chemical, and biological treatment approaches, wastewater remediation using microalgae is advantageous for many reasons. In addition to the known high growth rate of microalgae, the integration of microalgae in wastewater treatment also confers other environmental-related benefits, such as sustainable biomass production and carbon dioxide fixation [15,16,17]. Apart from these merits, microalgae-enabled wastewater treatment also offers an economical advantage in terms of the reduction of energy consumption relative to conventional treatment methods [18]. Microalgae-enabled treatment systems have been attempted for the removal of heavy metals [19], synthetic dyes [20], endocrine-disrupting chemicals [21], pharmaceuticals, and personal care products [22].

The increasing scientific attention for integrating microalgae with the wastewater system also lies in the abundant occurrence of organic substances and nutrients in many industrial wastewaters [23,24]. As the cultivation of microalgae requires considerable inputs of nitrogen and phosphorous, the up-taking of nutrients from wastewater culturing medium allows for the recovery of nutrients from various wastewater streams. Owing to their fast growth rates and high productivity, microalgae can efficiently obtain nutrients and use organic carbon that is present in wastewater [25]. Considering the cost of producing vital nutrients such as nitrogen and the decline in global reserves of some nutrients such as phosphorus, circulating nutrients from sewage streams through microalgae cultivation is an alternative for alleviating the bottlenecks in such scenarios by reducing the overall cost of biomass production. By closing the nutrient loop, the integration of microalgae can be used as a tool to bring circularity to the entire waste management and reutilization systems. The mechanisms of nutrient removal have been previously discussed [26]. The metabolic pathways of algal cells can be broadly distinguished by the four major elements, namely carbon, nitrogen, phosphorus, and sulfur. Nevertheless, primary attention is usually focused on nitrogen and phosphorus. The development and application of microalgae photobioreactors (PBRs) for wastewater treatment have been widely studied. The critical factors have been investigated to improve the wastewater treatment efficiencies, including: PBR modelling, design, and configurations; biomass generation and yield; and operating parameters and wastewater characteristics [27].

Membrane technology has been widely used for wastewater treatment, desalination, and resource recovery [28]. The integration of membrane technology and microalgae has been explored. Membranes are used for microalgae dewatering to allow for the conversion of the microalgal suspension into a thick slurry for harvesting [29]. Membrane-based separation techniques used for microalgae harvesting and by-product separation techniques based on the integration of various types of pressure-driven and osmotically driven membrane processes have been widely discussed [30]. The membrane technology’s high efficiency and low shear force features are interesting for force-sensitive algae as the process induces less disruption to the microalgae cells [31]. With the increasing awareness of waste reutilization, more efforts are delving into the development of membrane photobioreactors (MPBRs), a hybrid system consisting of microalgae and membrane filtration for simultaneous wastewater treatment and nutrient recovery. Compared with conventional large-scale microalgae-based wastewater treatment, i.e., open ponds and PBRs, MPBRs can significantly reduce the nutrient level in the wastewater culture medium and produce highly concentrated biomass. However, due to the integration of biological and physical treatment units, which impose greater complexities for their operation, the implementation of MPBR systems is confronted by several challenges [32]. The ongoing MPBR research efforts have been focused on optimizing the operating conditions and improving the performance of the membrane. Luo et al. discussed the important operating parameters of a submerged MPBR for microalgae cultivation and wastewater treatment. The parameters affecting the growth of microalgae and wastewater treatment efficiencies were evaluated, including the hydraulic retention time (HRT), solids retention time (SRT), aeration, and lighting [32]. Ye et al. reviewed the applications of several membrane integrated biological processes including an MPBR for nutrient recovery from wastewater, reject water, and sludge dewatering filtrate [33]. The coupling of membrane processes with a conventional photoreactor has been identified as an attractive strategy for overcoming the limitations of the traditionally used PBRs and ponds.

The concept of integrating membrane technology and microalgae for wastewater remediation has been established for quite some time, and many studies have been performed to assess the feasibility of the integration; nevertheless, the overview of research activities on this topic is still lacking. Particularly, recent innovations in MPBRs and membrane designs aimed at improving nutrient recovery and membrane fouling control have not been discussed. Given the current knowledge gaps, this review is set to provide an insight into the current development of membranes and membrane systems tailored for integration with microalgae-enabled wastewater treatment. The current challenges in implementing MPBR are identified, and the relevant recommendations are made to provide future research directions in a more practical and effective way.

## 2. Wastewater as a Source of Nutrients for Microalgae

Wastewater is a complex matrix that contains pollutants and also a range of reusable substances such as water, organic compounds, nutrients, and biosolids, which exist in dissolved or suspended forms. Nutrients found in wastewater, which are mostly compounds of carbon, nitrogen, and phosphorus, have been increasingly understood as an important constituent of wastewater [34,35]. Agricultural activities have considerably contributed to the surging amount of nitrogen and phosphorus in the receiving water bodies. Due to the extensive use of fertilizer in agricultural lands, majority of the reactive nitrogen compounds present in the fertilizers introduced are lost to the water bodies [33]. The removal of nitrogen and phosphorus during the wastewater treatment process is crucial, as the excessive amount of these nutrients can cause eutrophication, groundwater contamination, and deterioration of aquatic ecosystems [36]. Besides affecting the water quality and ecosystem balance, excessive consumption of nitrate and phosphorus has been associated with negative impacts on human health. For instance, nitrate is also known as a potential source of risk to human health [37]. Methemoglobinemia, thyroid difficulties, and diabetes are the commonly observed health hazards caused by excessive consumption of nitrate or nitrite compounds. The effective recovery of nutrients from the potential sources not only reduces the risk of exposure to the potential hazards but also provides an alternative for preventing the depletion of resources [38]. Nutrients such as nitrogen-containing compounds recovered from wastewater can be directly used as a fertilizer or can be further processed to form other commercially attractive products [39,40].

Anaerobic digestion has been widely used for the degradation of a range of organic wastes including agricultural and agro-food waste, wastewater sludge, municipal solid waste, and animal waste. Anaerobically treated effluents contain nutrient solutions with high concentrations of ammonium, nitrogen, and orthophosphate [41]. However, the removal of nitrogen from this effluent through a conventional wastewater plant is normally ineffective due to the low carbon/nitrogen ratio upon the consumption of organic carbon for the production of biogas and microbial biomass. Although the innovative bacterial nitrogen removal pathways involving nitrification/denitrification can be effectively used to remove nitrogen in a carbon-deficient condition, the process does not facilitate the recovery of nitrogen or other nutrients as new resources [42]. The nutrient-containing effluent is generally known as hygienically safe and odor-free, which means it can be directly used as a source of liquid fertilizer; despite this, the storage and transportation of a huge liquid volume has become the major constraint of their application in suburban-located agricultural fields.

**Figure 1:**
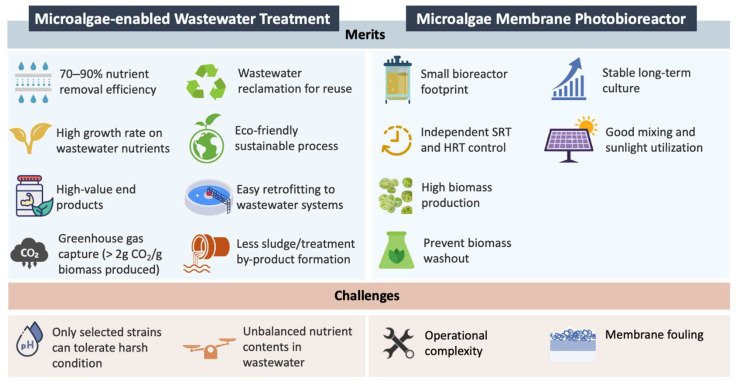
Summary of the merits and challenges of microalgae–enabled wastewater treatment.

Microalgae are self-sustained cells that hold vast potential for biomass production. They are also extensively explored for their therapeutic properties, which are beneficial for pharmaceutical applications. Microalgae obtain nutrients through a mixotrophy mode and can flexibly survive under extreme environmental conditions. These properties make microalgae a suitable candidate to efficiently uptake nitrogen and phosphorus and remove pollutants from a wide range of wastewater [43,44]. Furthermore, the organic compounds present in most wastewaters, such as carbohydrate and organic acids, can serve as a cheap source for microalgae growth [45]. The cultivation using microalgae such as Chlorella [46,47], Scenedesmus [48,49], and Phormidium to treat domestic and industrial wastewater have been widely reported. Particularly, Chlorella vulgaris has been extensively used for this purpose, owing to its high growth rate in diverse environments as well as its high adaptability towards high temperatures, wide pH ranges, and high salinities [50,51,52]. Through the interesting phycoremediation approach [53], microalgae can effectively treat organic pollutants that are known to be the major contaminants present in various waterways, hence reducing the chemical oxygen demand (COD) in wastewater [54,55]. While consuming the organic matters, the photosynthesis of microalgae also reduces carbon dioxide and increases dissolved oxygen. Unlike a conventional wastewater treatment plant, which mainly aims to reduce pollutants in wastewater to comply with the standard so that the treated water can be released into the environment without imposing significant adverse effects to the receiving waters, the integration of microalgae into wastewater plant offers additional advantages [56]. The cultivation of microalgae in nutrient-rich wastewater allows for the direct reutilization of nutrients from the medium. The integration of microalgae technology in wastewater treatment for nutrient recovery is also competitive over conventionally used anaerobic digestion and precipitation in terms of its carbon footprint and energy demands [57]. Figure 1 summarizes the merits and challenges of microalgae-enabled wastewater treatment [58,59,60,61,62].

The efficiency of nutrient recovery depends on both the process conditions and nutrients themselves. The load, concentration, and chemical compounds of the nutrients must be taken into consideration when evaluating the nutrient potential of a wastewater stream [63]. Many commonly produced wastewaters, such as wastewater from municipal sources, dairy manure, and swine wastewater contain nitrogen to phosphorus molar ratios that are suitable for the growth of microalgae [64]. Typical sewage and agricultural wastewater contain 15–90 mg/L of nitrogen and 4–20 mg/L of phosphorus, which can sufficiently serve as a cultivation medium to support algal growth [60,65]. The biomass of microalgae is a crucial factor in algae-based wastewater remediation and nutrient recovery, where the higher the biomass, the higher the removal efficiency. In general, microalgae can consume nutrients through various mechanisms to generate biomass. Nitrification is the major route of nitrogen acquisition by green microalgae, where the ammonium compounds are used to react with oxygen to form NO_2_^−^ and NO_3_^−^. On the other hand, biomass adsorption and precipitation are the two main phosphorus removal mechanisms in microalgal systems [66]. The removal of phosphorus in a microalgae-PBR is mainly through the precipitation of calcium phosphate, which is facilitated by the microalgae [67]. Different species of algae exhibit different abilities and capacities in removing nutrients [68]. In addition, the nutrient removal efficiency is also governed by external factors such as operating and seasonal conditions [69,70]. The pH of the suspension and surface charge of the biomass considerably affect the nutrient removal efficiency; the extent of the efficiency depends on the type of nutrients present and their removal efficiency [71]. In an alkaline solution, the predominant forms of phosphate ions, HPO_4_^2−^ and PO_4_^3−^, encounter strong electrostatic repulsions that interfere with the adsorption of these ions on the highly charged microalgae surface [72]. Therefore, maintaining a low pH would be beneficial for the adsorption of orthophosphates by the microalgae and for the enhancement of phosphorus removal through the formation and precipitation of complex salts in the wastewater. In the high pH condition, the transformation of NH_4_^+^ to NH_3_ would be promoted, which increases the stripping of nitrogen into atmosphere.

The main challenges in algae-based wastewater remediation are the fluctuating composition and unbalanced ratio of major nutrients, including carbon, nitrogen, and phosphorus; the presence of some compounds that can disrupt the biomass production and cause inefficient nutrient removal in wastewater treatments is also a challenge [73]. The nutrients present in varied chemical forms in wastewater are utilized by microalgae to different extents. The consumption of ammoniacal nitrogen (NH_4_^+^-N) by microalgae requires less energy, as NH_4_^+^-N can be directly absorbed to produce amino acids [74,75]. Nitrate (NO_3_^−^-N) can only be assimilated after several cycles of reductions and hence is less favorable. External parameters such as light conditions, additional carbon dioxide supply, or extended HRT are necessary for boosting the photosynthetic process, thus enhancing the NO_3_^—^N removal [76]. However, the high uptake of NH_4_^+^-N does not necessarily promote cell growth; this has been indicated in some studies where Scenedesmus bijugatus and Monoraphidium sp. were observed to produce greater biomass when fed in a NO_3_^−^-N -rich medium [77,78]. In addition to the growth rate and biomass amount, the removal of nitrogen can also be associated with the uptake of phosphorus, which has been considered to be a growth-limiting substrate in wastewater treatment applications. Phosphorous deficiency due to the loss of phosphorus via precipitation would impose a negative effect on nitrogen removal [79]. Several approaches have also been established to facilitate the acclimation of microalgae in wastewater, which include the selection of wastewater-tolerant species, mixed cultivation of two or more microalgae [80], mixing of wastewater and synthetic culture medium, or using different types of wastewater [81,82].

The high-rate algal pond, an integrated system that treats wastewater in low energy conditions, has been widely used for providing secondary and partial tertiary-level treatments in large volumes [83]. Despite incorporating many attractive features over conventional treatment plants, high-rate algal ponds require shallow depths for sunlight harvesting and relatively long HRTs for efficient treatment. Therefore, the system has been associated with the major disadvantage of a large areal footprint [84]. On the other hand, biological nutrient removal processes have been conventionally implemented for the removal of total nitrogen and/or total phosphorus. While exhibiting high efficiency in nutrient removal to meet the discharge standard, biological nutrient removal processes have been unfavorably associated with some limitations, specifically in terms of process complexity and cost. With the increasing demands for utilizing microalgae or co-cultivated microalgae-bacteria for nutrient recovery, the MPBR has been developed as a potential candidate for delivering the desired outcome without compromising the technical issues of footprint, cost, and complexity in upscaling and retrofitting. The nutrient removal efficiency in the system is mainly caused by the nutrient uptake via microalgae instead of through membrane filtration.

## 3. Microalgae Membrane Photobioreactor

Microalgae can be cultured in open and closed systems [85]. The combination of microalgae cultivation and membrane technology in a closed system forms an MPBR system, which demonstrates several advantages in treating wastewater compared with conventional MBRs. In addition to addressing the issue of conventional algae ponds commonly suffering from low efficiency due to insufficient light penetration and low biomass generation, MPBRs offer smaller bioreactor footprints and can be continuously operated at low HRTs. The SRT and HRT can be independently controlled in an MPBR system to increase the nutrient load without requiring a large land area for the cultivation of microalgae [86]. The decoupling of HRT and SRT by membrane filtration could result in a doubled biomass productivity and several-fold higher recovery rate for nitrogen and phosphorus in MPBRs compared with conventional PBRs [87]. Biomass washout, a major technical issue associated with microalgae cultivation during continuous operation, can be overcome in MPBRs [88]. Due to the issue of maintaining slow algal growth with short HRTs in conventional reactors, it is required to increase the biomass concentration or reactor volume to achieve the desired level of nutrient removal. With the presence of a membrane for solid–liquid separation in the MBR, it is possible to achieve a short HRT bound to a long SRT so that the biomass concentration can be increased. As the filtration mechanisms rendered by membrane enables MPBRs to operate with higher supply rates, the biomass yield and nutrient recovery efficiency are significantly higher in MPBRs compared with PBRs [89].

Progress has been made in the development of MPBRs in terms of their construction and operations. The membrane process has been known as a versatile technique for treating a wide range of wastewater, but it is difficult to identify a single membrane process that fits all purposes. The integration between membrane technologies and microalgae cultivation enables the reclamation of essential resources including water, nitrogen, and phosphorus from wastewater. When integrated in a microalgae photoreactor system, membranes can be configured into crossflow, dynamic, submerged, and forward osmosis (FO) modes [90]. The appropriateness of an identified membrane process relies on the nature of wastewater and setup of the photoreactor. These configurations demonstrate the advantages and limitations, which in turn dictate the efficiency of the membrane processes in dealing with wastewater treatment and nutrient recovery. Similar to how the species of microalgae can considerably affect the growth rate and yield, the selection of a compatible membrane process is crucial in determining the overall performance of the MBPR [91]. The membranes in MPBRs play an important role in separating biopolymers such as proteins and carbohydrates from the permeate; however, the removal efficiency is strongly governed by the molecular weight cut-off of the membranes. Each type of membrane process exhibits unique characteristics in wastewater remediation but is also coupled with their own merits and flaws. The classical type of MPBR consists of a membrane submerged into the reactor [92]. Carbon dioxide and illumination are supplied to sustain the growth of microalgae. Aeration is also required to scour the membrane surface for fouling control. The membranes typically used in MPBR are microfiltration and ultrafiltration that are arranged in flat sheet or hollow fiber configurations. Different light sources can be applied for the cultivation of microalgae in MPBRs. While outdoor direct sunlight illumination represents the most realistic condition, the supply is inconsistent, and the intensity fluctuates throughout the day. Therefore, most lab settings are equipped with artificial light sources, such as fluorescent and multi-LED lights.

Despite the capability and reliability of producing high-quality product water, pressure-driven processes are known as energy-intensive processes that would impede the attainment of economic advantage. Therefore, there has been increasing attention in developing membrane processes that are osmotically driven to reduce the energy requirement [93]. Akin to natural osmosis, FO has been promoted as an energy-advantageous alternative to reverse osmosis (RO). Driven by the osmotic pressure difference between the feed water and the draw solution, water can be transported across the FO membrane from the feed water with a lower osmotic pressure to the draw solution with a higher osmotic pressure. FO is a promising candidate for treating complex wastewater without the requirement of sophisticated pre-treatment on account of their low fouling propensity [94]. Similar to typical pressure-driven membrane processes, FO can be configured into several ways depending on the type of wastewater to be treated [95]. An MPBR that operates with an FO membrane, also known as an osmotic membrane photobioreactor (OMPBR) exhibits high solute rejection, low fouling tendency, and high durability when dealing with complex wastewater [96,97]. The promising performances of osmotic membrane bioreactors (OMBRs) have been witnessed in a wide range of biological wastewater treatment applications and are evident in the nutrient recoveries obtained from wastewater [98,99,100].

Many key components must be considered when implementing the newly established system, especially for a full commercial scale. One of the most cost-effective methods for increasing wastewater treatment efficiency while achieving high biomass productivity is through the manipulation of operating conditions [101,102]. Similar to an MBR, the efficiency of an MPBR is largely governed by the SRT and HRT. The former is a critical factor that controls biomass concentration and productivity as well as nutrient uptake by the microalgae. Compared with heterotrophic bacteria with high metabolic rates, a longer retention time is required for the microalgal biomass to effectively absorb nutrients. The regulation of SRT, HRT, and SRT/HRT ratio is necessary to maximize algal productivity and nutrient uptake by the microalgae cultured in wastewater. However, the SRT and HRT required to achieve the highest biomass yield and the highest nutrient removal efficiency are commonly not in coincidence. Therefore, most studies have adopted a moderate SRT and HRT for the operation of MPBRs [70,103]. An MPBR operating with a long SRT is not favorable for nitrogen removal, as the long SRT results in less biomass waste [67]. The STR also influences the removal efficiency of phosphorus, as the change in the microalgal biomass concentration would affect the algae-assisted chemical precipitation of phosphorus. In any bioreactor, the HRT controls the nutrient loading and treatment capacity. The HRT affects the biomass concentration and solid–liquid separation efficiency in MPBRs. In addition to the SRT and HRT, the organic strength of the wastewater and initial concentration of the microalgae also play critical roles in determining the treatment efficiency.

## 4. Performances of Microalgae-MPBR for Wastewater Treatment and Nutrient Recovery

**Figure 2:**
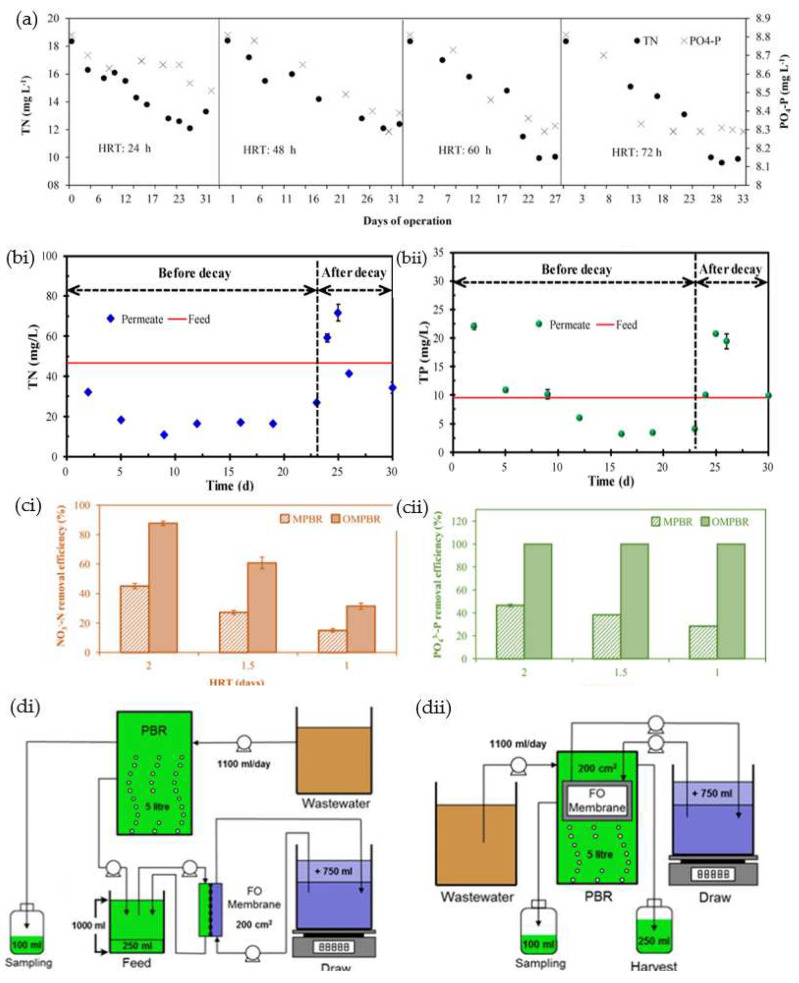
(**a**) Concentrations of total nitrogen and PO_4_^3^P in the effluent of the MPBR operated with different HRTs [104]. (**b**) Concentration of (i) total nitrogen and (ii) total phosphorus before and after the decay of microalgae in the MPBR operated under a long HRT [105]. (**c**) Removal efficiencies of (i) NO_3_^−^-N and (ii) PO_4_^3−^-P in the MPBR and OMPBR as a function of hydraulic retention time [110]. (**d**) Illustrations of (i) sidestream and (ii) submerged FO modules in the OMPBR [111] (Reprinted with permission).

The wastewater treatment and nutrient removal efficiency of MPBRs have been evaluated as a function of the key operating parameters. By using the mixed microalgae of *Chaetophora* sp. and *Navicula* sp. cultured in a synthetic secondary treatment effluent, Solmaz and Işık investigated the effect of the HRT on nutrient removal rate while maintaining an SRT of 3 days [104]. With an HRT of 24 h, the removal rates of total nitrogen and PO_4_–P were reported as 5.55 mgL^−1^day^−1^ and 0.40 mgL^−1^day^−1^, respectively, where the highest biomass production rate was obtained. As shown in Figure 2a, the prolonged HRT was beneficial for a better nutrient removal performance due to better nutrient assimilation by the microalgae. Zou et al. evaluated the feasibility of an MPBR system for municipal wastewater treatment under long-term operation with a high SRT of 50 days [105]. The decaying of microalgae on the 23rd day implied that the MPBR system could not sustain the performance due to the trade-off between the biomass concentration and light penetration. Before the occurrence of microalgae decay, the MPBR achieved nitrogen and phosphorus removal efficiencies of 76.7% and 66.2%, respectively. However, as shown in Figure 2b, the decay of microalgae resulted in a dramatic increase in the concentration of total nitrogen and phosphorus due to the release of cytoplasms from microalgae decomposition.

An outdoor pilot-scale MPBR which coupled a hollow fiber ultrafiltration membrane system with *Scenedesmus* sp. was developed by Viruela et al. to treat effluents from an anaerobic MBR sewage treatment [106]. With an SRT of 4.5 days, the MPBR achieved PO_4_^3−^-P and NH_4_-N removal rates of 1.17 mg/L.day and 7.68 mg/L.day, respectively. Although it was expected that the nitrogen to phosphorus influent ratio could affect the nutrient uptake by the microalgae, the statistical correlations for long-term operation have yet to be established. By manipulating the outdoor environment and operating condition, it was observed that high biomass concentration, reduced solar irradiance, and temperature higher than 25 °C had negative effects on the nutrient uptake efficiency and biomass productivity. It was also revealed that the pumping and recirculation modes can be further optimized to reduce the energy demands and footprints while maintaining the nutrient recovery rate and biomass productivity. The optimization of the HRT had insignificant effects on the photosynthetic efficiency and nutrient recovery rates [107]. However, a prolonged SRT of up to 9 days dramatically increased the membrane fouling rate due to the high biomass concentration. The intensity and path distance of light directly affect the photosynthetic efficiency of microalgae. By varying the light path distance, an improvement in the photosynthetic efficiency of mixed microalgae culture of Chlorella vulgaris and Scenedesmus was observed. Using the same outdoor pilot-scale MPBR, González-Camejo et al. reported an increase in the nitrogen and phosphorus recovery rates by 150% and 103% respectively; microalgae biomass productivity increased by 194% and photosynthetic efficiency by 67% when the light path distance was reduced from 25 to 10 cm due to a better sunlight harvesting [108].

Preveen and Loh operated an OMPBR for 162 days for a tertiary wastewater treatment and nutrient recovery scenario using *Chlorella vulgaris* [109]; Removal efficiencies of 93% 53% and 89% were achieved for NH_4_^+^-N*,* NO_3_^−^-N, and PO_4_^3−^-P at an HRT of 3 days. A high tendency of microalgae aggregation and attachment to the bioreactor and membrane surfaces was observed, resulting in the accumulated total biomass in the OMPBR being over 5 g/L. By changing the composition of the wastewater, it was noted that the OMPBR when operated at high nitrogen and phosphorus concentrations resulted in a high accumulation of nutrients during the transient stage, where the biomass and nutrient assimilation was still low. Such an observation suggested that an OMPBR would be more sustainable for tertiary wastewater treatment with low nitrogen and phosphorus contents. The efficiencies of microfiltration and FO in an MPBR operated with Chlorella vulgaris for continuous tertiary wastewater treatment have been compared [110]. While both photoreactors exhibited a comparable biomass accumulation of >2 g/L, the OMPBR consistently achieved higher nutrient removal efficiencies regardless of the operating conditions, on account of the higher solute retention properties of the FO membrane. The concentrations of NH_4_^+^-N, NO_3_^−^-N, and PO_4_^3−^-P were significantly reduced due to the uptake by microalgae. As shown in Figure 2c, the OMBPR achieved higher removal efficiencies of up to 99% and 100% for nitrogen and phosphorus, respectively, compared with the MPBR efficiencies of 97% and 46%, respectively. Nevertheless, due to the fundamental differences in microfiltration and FO—in which draw solution cost and its regeneration cost must be considered for the latter case—the OMPBR displayed a higher operating cost and overall filtration cost relative to the MPBR. However, FO may stand a better chance to energetically outperform the conventional MPBR if the photoreactor was operated at a higher flux and for a longer operating duration, where the transmembrane pressure and fouling become more significant in MPBR.

The effects of sidestream and submerged FO module configurations on nutrient removal efficiency and microalgal growth have been evaluated, as illustrated in Figure 2d [111]. A higher algae biomass was obtained in the submerged OMPBR, hence leading to a higher nutrient removal efficiency of 100% for NO_3_^−^-N and 92.9% for PO_4_^3−^-P compared with the sidestream counterpart, which had a removal efficiency of 96% for NO_3_^−^-N and 82% for PO_4_^3−^-P. Due to the higher initial water flux in the sidestream OMPBR, severe flux loss and greater foulant deposition were observed. However, because a more convenient hydraulic flushing could be performed on the sidestream module relative to the submerged one, the former is more favorable when dealing with complex wastewater that imposes a high fouling tendency in the OMPBR.

## 5. Membrane Fouling in Microalgae–Wastewater Medium

While the efficiencies and simplicity of membrane-based processes for wastewater treatment have been well-proven, the process is confronted by membrane fouling, a process whereby soluble and particulate materials attach onto the membrane surface or adhere to the membrane’s pores. As the efficiency of membrane filtration deteriorates over time due to membrane fouling, this phenomenon has been identified as the main contributor of the total operational cost in MPBRs. For a membrane system operated under low hydraulic pressure, polysaccharides and other biopolymers are known as the major foulants that contribute to membrane fouling. The main contributors of fouling in the MPBR are organic substances and microalgal biomass, while the fouling mechanisms are similar to that of the conventional MBR [112]. When integrated into the wastewater treatment system, the fouling of the membrane, which has been known to be the impediment of any membrane-based separation process, is not only caused by the microalgal suspension, algal organic matters (AOM), and cellular debris, but also other fractions derived from the effluents, such as organic substances and microorganisms [113]. The AOM released by microalgae are characterized by different molecular weights and chemical compositions, which depends on the algae species and their respective growth phase as well as the nutrient availability. Specifically, external organic matters (EOM) and internal organic matters (IOM) are the terms used to differentiate the AOM produced from the metabolic activities and those released from the cell rupture of microalgae cells, respectively.

**Figure 3:**
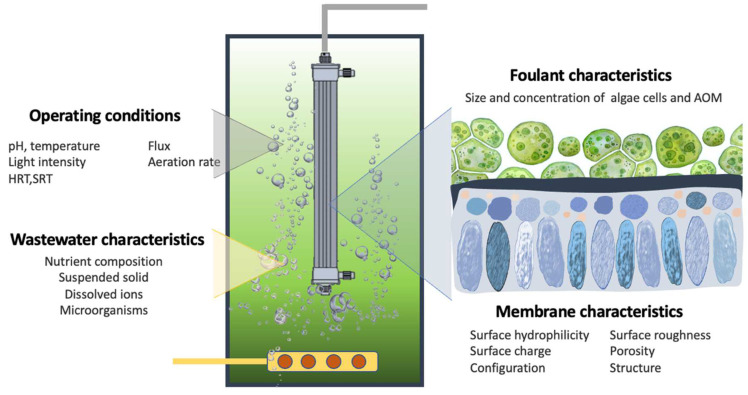
The major fouling-contributing components during wastewater treatment in microalgae-containing MPBRs.

Microalgal cultures have been identified as a major contributor of initial fouling; however, the supernatant contents, including EOM and cellular debris, can considerably change the fouling behaviors. The membrane fouling behaviors in the presence of a microalgal solution are governed by many factors, including the driving force of the membrane processes, the operational modes, and the interactions among the membrane surface, microalgae, and other components surrounding them. The surface properties of the membranes such as the surface hydrophilicity/hydrophobicity, surface roughness, and surface charge dictate the interactions between the membrane surface and foulants and hence the depositions of various foulants on the membrane structure. Fouling takes place through different mechanisms and with different severity in various membrane processes. It is generally agreed that membrane fouling in osmotically driven processes such as FO is less severe and is normally reversible. It has also been observed that the carbohydrates and proteins of soluble microalgae products fouled an FO membrane to different extents when operated in active layer facing feed solution and active layer facing draw solution configurations [114]. The interaction between the soluble microalgae products and calcium-containing draw solutions could further increase the severity of membrane fouling. Figure 3 illustrates the intercorrelated fouling-contributing components during wastewater treatment in microalgae-containing MPBRs.

Understanding of the fouling types, mechanisms, and governing factors during the filtration process is important for mitigating the issue through the optimization of membrane designs and operating conditions. Membrane microalgae fouling initiates with the deposition and accumulation of microalgal organic matters, algal cells, and transparent exopolymer particles (TEP). The process can be further elaborated as a multiple-stage process, which involves pore-blocking, gel-layer formation, multicellular algal complex cake layer formation, and the random distribution of foulants throughout the cake layer and membrane surface [115]. Complex fouling mechanisms take place during the filtration of microalgae cultured in wastewater medium. Membrane pore blocking and cake layer formation on the membrane surface are mainly caused by the microalgal cells and EOM that are excreted as metabolism products or readily exist in the wastewater. EOM with small molecular size can easily penetrate into membrane pores and form gel layers on the membrane surface, whereas the microalgal cells tend to build up as a cake layer on the membrane surface. While the deposition of EOM leads to an irreversible fouling resistance, the fouling caused by the cake layer build-up is normally reversible and can be removed by physical cleaning procedures such as online or intermittent backflushing. In addition to physical cleaning, mechanical and chemical cleaning methods have also been adapted to restore membrane performance [116]. Chemical cleaning, which involves the soaking of the membrane in a cleaning chemical, has been commonly applied to address irreversible fouling. Regardless of the approaches used, the efficiency of the membrane cleaning depends on multiple factors, such as the fouling nature, extent of fouling, and cleaning frequency.

Using model secondary effluent wastewater, Lee et al. observed that TEP played an important role in the initial stage of biofilm formation and in the biofouling mechanisms of the RO membranes [117]. The cake fouling potential of TEP significantly increased the bacterial deposition on the biofouled membrane. Desmodesmus sp. and Coelastrella sp. have been cultured in a submerged membrane-based filtration device using anaerobic membrane bioreactor-treated secondary effluent as the culturing medium [118]. Based on the different fractions of the microalgal suspension, it was observed that the fouling mechanisms and fouling reversibility of the polyvinylidene fluoride (PVDF) ultrafiltration membrane were strongly dependent on the stages of microalgal growth. Irreversible fouling took place in the early stage of the filtration process; interestingly, however, the interactions among the microalgae cells, cell debris, and EOM in the suspension altered the fouling behaviors, and the effect of reversible fouling was decreased as the filtration duration lapsed. Nevertheless, compared with the normal culture medium, the high organic load of the anaerobic membrane bioreactor treated secondary effluent led to a very high fouling propensity.

The rupture of microalgae cells and their bindings with polymeric substances could change the surface chemistry and structure of the cells as well as their interaction with membrane surface. As the organic carbon contents present in most wastewater are known to be major foulants, the total organic content should be kept minimum. The elimination of organic carbon content from wastewater prior to its usage is beneficial for minimizing membrane fouling in the integrated system. The control of the release of EOM during microalgae cultivation by optimizing the environmental conditions such as the pH, temperature, and culture stage can also suppress the fouling propensity [119]. The effective control of TEP through pre-treatment is also essential for controlling the propagation of biofouling. The microalgae concentration has a considerable effect on the severity of membrane fouling. An increased viscosity in the bulk medium was observed to hamper the air scouring along the membrane surface [120].

Using synthetic wastewater that was rich in nitrogen in the forms of NH_4_^+^-N and NO_3_^−^-N, Luo et al. observed the effects of nitrogen chemical compositions on the biomass growth rate and fouling propensity of submerged PVDF hollow fiber membranes [121]. The MPBR fed with NH_4_^+^-N-rich wastewater produced a greater amount of extracellular materials and had poorer dewaterability compared with the NO_3_^−^-N-fed counterpart. With NH_4_^+^-N present as a preferable source of nutrient under the aerobic condition, the growth and accumulation of bacteria were stimulated. At high transmembrane pressure where the flux was above 25 L/m^2^·h^1^, the biomass cultured in NH_4_^+^-N-rich wastewater caused more rapid fouling than the NO_3_^−^-N-fed counterpart due to the formation of a high concentration of biopolymer. The production of biopolymers resulted in heterogeneity and increased the biomass hydrophobicity, hence forming a more severe bio-cake on the membrane surface. As such, it was recommended that the SRT should be shortened to control the biomass heterogeneity when treating NH_4_-rich wastewater. The findings also implied that the composition of nitrogen in the wastewater should be examined so that the operating conditions can be tuned to mitigate fouling and reduce the harvesting cost. Zhang et al. studied the membrane fouling behavior in an MPBR that treated a synthetic anaerobic digestion effluent [122]. It was observed that the SRT significantly affected the extent of membrane fouling, but in a nonlinear correlation. By determining the filtration resistance, gel layer formation and pore clogging were identified as the main contributors to the membrane fouling. At an SRT of 20 days in which the extracellular polymeric substances (EPS) and soluble microbial contents were at their maximum level, the fouling was further worsened.

For wastewater with highly concentrated organic loads, the growth of microalgal cells and the subsequent biomass production are inhibited by the microalgae products, which are mainly composed of carbohydrates and proteins. To counter this issue, the co-cultivation of microalgae and microorganisms such as bacteria in algae-bacterial symbiotic systems has become the prevailing method for improving the microalgae growth rate and treatment efficiency of wastewaters. Being increasingly used in wastewater treatment, the microalgal–bacterial consortium composed of activated sludge, algae, and bacteria demonstrate symbiotic interactions that are beneficial for promoting higher nutrient removal efficiency as compared to the single counterpart [123,124]. Amini et al. achieved NH_4_^+^-N and PO_4_^3−^-P removal efficiencies of 94% and 80%, respectively, using microalgae and activated sludge inoculum ratios of 5:1 in a semi-continuous MPBR [125]. With the presence of microalgae in the MPBR, it has been reported that the mechanical aeration required for floc agitation and membrane cleaning was reduced by 60% compared with a conventional bioreactor with only activated sludge biomass. By controlling the mechanical aeration, the stable growth of algae and bacteria can be achieved to increase nutrient removal efficiency. Although it was predicted that the energy cost can be reduced by 36% by reducing the mechanical aeration, the cost of energy required for the light supplied was not considered in the study [126]. The formation of more porous and layered channels in the fouling layer of the co-culture of algae and activated sludge has been observed, implying that the co-cultivation can reduce the severity of membrane fouling [127]. However, using microalgal-activated sludge co-cultivation at the optimized ratio for raw wastewater treatment, Chaleshtori et al. reported severe membrane fouling despite the high nutrient removal efficiency due to the spike in carbohydrate and protein fractions in the soluble microbial products and EPS, respectively [128]. 

## 6. Innovations in Membranes and Reactor Design for MPBR

**Figure 4:**
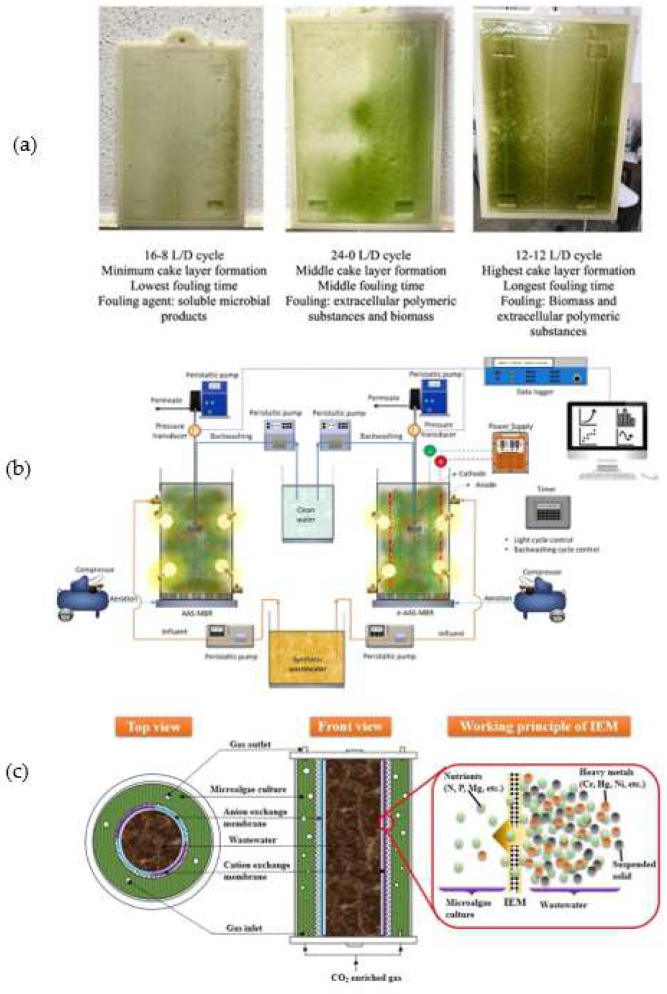
(**a**) The appearance of membranes operated in various light/dark (L/D) conditions. (**b**) Schematic illustration of conventional and electrically induced microalgae-activated sludge MPBR [133]. (**c**) Annular MPBR equipped with ion exchange membrane to separate microalgae cultivation and nutrient-containing wastewater [134] (Reprinted with permission).

Several approaches have been accommodated in microalgae-based MPBRs to improve nutrient recovery and wastewater treatment efficiency. In addition to maximizing the performance of MPBRs, the mitigation of membrane fouling is always a primary task in membrane research. The periodical membrane backwashing in the MPBR system has been proven effective in achieving a high permeate flux recovery of >80% [129]. Designs of an antifouling membrane and innovative reactor have also been made to suppress the membrane fouling tendency in MPBRs. In situ mechanical cleaning of membrane in the MPBR has been established to mitigate fouling during operation and reduce the consumption of chemical agent. Azizi et al. developed a reciprocal MPBR that includes a spongy blade for the cleaning of the cake layer formed on the membrane surface [130]. A programmed PLC system was designed to enable the detection of transmembrane pressure (TMP) of the MPBR membrane so that the spongy blade can be activated for cleaning. The mechanical cleaning reduced the total hydraulic resistance by up to 83% without the need for chemical cleaning or washing. As shown in Figure 4a, it was observed that the dark–light operational periods affected the severity of the cake layer formation, time taken to cause detectable change in TMP, and major foulants in the MPBR. Hosseini et al. installed orifices with different diameters to alleviate the cake resistance and pore blocking resistance in an MPBR containing spirulina [131]. Polyethylene granular particles with a diameter of 5mm were loaded into the MPBR. The granules penetrated through the boundary layer of the membrane and removed the cake layer deposited on the membrane.

A nanocomposite membrane is a cutting-edge innovation in membrane development. Enabled by the surface functionalities and structural advantage of nanomaterials, the resultant nanocomposite membranes exhibit enhanced efficiency in wastewater treatment. Chong et al. fabricated silver/graphene oxide (Ag/GO)-incorporated PVDF membranes for the treatment of synthetic municipal wastewater [132]. In the presence of Chlorella vulgaris, the nanocomposite membrane with increased surface hydrophilicity not only increased the water permeability, but also contributed to a better anti-fouling propensity, especially for long-period operation. The reactive oxygen species generated by the Ag/GO nanohybrid rendered strong antimicrobial properties on the membrane surface, thus preventing the attachment of E. coli, the commonly found microorganisms in typical municipal wastewater. The negligible difference in the biomass cultivated using commercial PVDF and Ag/GO incorporated PVDF implied that the nanomaterials have marginal effects on the microalgal growth. However, it is worth mentioning that disruption of microalgal cells by metal and metal oxide nanoparticles at high concentrations has been reported in some studies [133,134], suggesting that the loading of nanomaterials is an important parameter for the preparation of nanocomposite membranes.

The integration of electrochemical processes in PBRs has been proven to stimulate the growth of microalgae [135], improve nitrogen and phosphorus removal efficiency [136], and mitigate membrane fouling [137]. An MPBR incorporated with a low-voltage direct current was developed for the treatment of synthetic municipal wastewater using Chlorella vulgaris [138]. While achieving a biomass production that was comparable to the conventional MPBR, the electrokinetic-assisted MPBR achieved a significantly higher phosphorous removal, with an overall removal of 97.98% compared with the 41.81% achieved by the conventional MPBR. In addition to the typical adsorption and precipitation mechanisms, the electrochemical reactions taking place in the suspension and the ionic strength of the solution also contributed to phosphorus removal. The reduced phosphate concentration in the electrokinetic-assisted MPBR is advantageous for membrane fouling mitigation, as a low concentration of phosphorus retards the growth of biofilm. Despite the advantage of electrochemical reaction in stimulating phosphorus removal and subsequently controlling biofilm formation, an overall lower nitrogen removal has been observed, which suggests that the introduction of an electric field imposed an inhibitory effect on the removal efficiency of total nitrogen. A contradictory observation was reported by Corpuz et al., who treated synthetic municipal wastewater using Chlorella vulgaris-activated sludge [139]. Using an electrically induced MPBR as schematically shown in Figure 4b, the system not only achieved improved phosphorus removal by 65%, but also a 16.7% increase in NH_4_^+^-N removal as compared with the non-electro counterpart. The improvement in nitrogen removal efficiency has been attributed to several reasons: an accelerated denitrification process that resulted from the anoxic conditions induced by the electric field; and an electroreduction of the nitrate on the cathode and adsorption of nitrate into the electrocoagulated aluminum hydroxide. The applied electric field also reduced the contents of the membrane fouling precursor through several mechanisms. The negatively charged polysaccharides present in the microalgal suspension were neutralized by aluminum ions, meaning that their concentration as one of the major foulants in the bulk solution was reduced. EPS was decomposed via electrochemical oxidation into substances with low molecular weight and can be readily biodegraded. The inhibition of microalgae growth has been observed upon prolonged electric field exposure. However, no agreement has been made on the duration in which the retardation of microalgae starts to occur. As the retardation is mainly caused by the electrochemical reactions that take place at the electrode, the design of the rectors and the position of the electrodes can be further fine-tuned to minimize the oxidation effects.

An annular MPBR equipped with an ion exchange membrane has been developed to control the effects of suspended solids, nutrients, and heavy metals in wastewater on microalgal growth [140]. As shown in Figure 4b, the ion exchange membrane separates wastewater and microalgae into two chambers, hence preventing them from directly contacting each other. Nitrate and phosphorus from the untreated tannery wastewater were allowed to penetrate through the membrane into the microalgae culture, while the undesired components in wastewater were retained. Particularly, the high turbidity of wastewater, which reduces light penetration for the photosynthetic growth of microalgae can be avoided in the microalgae culture medium. In addition to a better microalgal growth in the ion exchange MPBR, higher nutrient recovery efficiencies have been observed for nitrate and phosphorus, which were 8.95 and 2.31 mg/L.d, respectively, compared with that of the conventional PBR, which had removal rates of 4.88 and 0.94 mg/L.d, respectively. However, despite the improvement made, the transport mechanisms of nutrients across the ion exchange membrane were not described in detail.

## 7. Challenges and Future Directions

**Figure 5:**
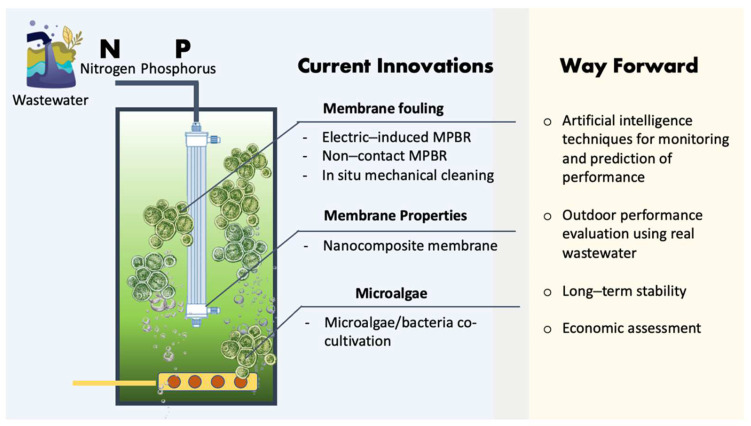
Summary of current innovations and the way forward for the implementation of MPBRs in wastewater treatment and nutrient recovery applications.

While microalgae have been more popularly known as a feedstock for biofuel production, current efforts have also been diverted to the application of microalgae for wastewater treatment and nutrient recovery. Microalgae-enabled wastewater remediation processes have demonstrated many benefits in meeting the new expectations for improved wastewater treatment, which also include bioremediation and nutrient recovery. As summarized in Table 1, studies in support of this claim have been published to prove microalgae-enabled MPBRs as a potential strategy for future wastewater treatment. The technical feasibility in terms of wastewater treatment efficiency has been well-observed through various experimental studies. Nevertheless, this relatively innovative approach is still confronted by many challenges, such as the adaptability of microalgae in complex wastewater as well as the design and optimization of processes to improve treatment efficiencies with a lower cost. In addition to these general challenges, the specific constraints can also be related to the applications of the membranes in the photobioreactor or processes involving microalgae-enabled wastewater treatment and nutrient recovery. Despite the efforts made in this field, many uncertainties remain unresolved. This also calls for the need to expand and deepen the research investigating the details of the influencing parameters. Due to the multiple factors involved in the process, more investigations are required before the underlying reasons that contribute to the treatment and removal of nutrients in the system can be pinpointed when compared with the conventional counterparts. Figure 5 summarizes the current innovations and the way forward for the implementation of MPBRs for wastewater treatment and nutrient recovery.

As a complex physical and biological integrated system, the nutrient recovery and wastewater treatment efficiency of MPBRs are subject to many factors as well as the operating conditions. The presence of microalgae further complicates the operation of an MBR that involves biological and physical treatment processes. Therefore, the quantification and optimization of the process can be considered as an important area of research in this field. Despite the investigations made in optimizing the operating parameters of MPBRs, such as examining the effects of HRT, SRT, and initial biomass concentration, there is still no consensus on the optimum operating conditions for MPBR operations. For instance, the HRT for the optimum microalgae productivity has been reported in a wide range, from one to several days. This indicates that the important operating parameters of the bioreactor, such as the SRT and HRT, are also highly influenced by other factors, such as the type of wastewater and microalgal species. The prediction of the behaviors of MPBRs in terms of nutrient removal efficiency and fouling propensity can be helpful in decision-making to improve the performance of the system. A systematic tool for optimization and prediction is required for this purpose. Very recently, machine learning models have been applied in predicting the nutrient removal efficiency of MBRs in treating sewage water, which investigated different modelling scenarios in various operating conditions [141]. The same efforts can be extended to MPBR applications to correlate the relationship between the operating parameters and the wastewater treatment or nutrient recovery performances. Similarly, artificial intelligence techniques, which can model real-time issues involving details of perplexing conditions, can play an interesting role in determining the quality of source water as well as in predicting the membrane filtration efficiency and membrane fouling [142].

Despite the tremendous efforts made in fouling controls, the actual conditions and parameters of the wastewater–microalgae–membrane-integrated system have not been extensively studied. The long-term stability of the membranes in such a complex environment has also been overlooked. Special attention should be paid to analyzing the foulant interactions and fouling behavior by working on the filtration of microalgae-treated effluents. Some of the adopted spectrometric or microscopic characterization tools only reveal semi-quantitative information, where important details such as the chemical compositional change during the propagation of fouling remain as a black box. More powerful tools and detection methods, which can also be feasibly utilized in real-scale membrane practices, are desired to evaluate the fouling potential of various microalgal organic matters. Furthermore, the prediction and assessment of the fouling behavior through some relevant indication such as a cake fouling index also deserves more extensive research effort. Due to the uncertainty in determining the chemical composition of naturally occurring microalgal organic matters, most microalgal biofouling studies were conducted using compounds that mimic the properties of the identified microalgal organic matters.

It is crucial to choose the suitable algal strain for an identified wastewater. There are multiple factors to consider, from the growth rate, nutrient consumption, and biomass production to their flexibility to adapt to the harsh nature of most wastewater. Although a good number of microalgae of different biochemical compositions has been proposed for wastewater treatment, when MPBR is of concern, the interactions between the microalgal cells and the membrane surface have not been thoroughly investigated. Other than the most studied Chlorella vulgaris, many other important microalgae strains can be considered for wastewater treatment [143]. Ensuring the presence of only a specific type of microalgae culture throughout the operation of PMBR is almost impossible. Therefore, studying the effect of co-existing microalgae in the receiving wastewater on the performance of the MPBRs is an interesting subject to achieve the targeted removal efficiency in practical conditions. Studies should also be focused on using real wastewater from primary and secondary effluents instead of synthetic wastewater. Real wastewater can provide a direct indication of the growth behavior of microalgae and their effects on membrane fouling during the bioremediation process. Co-cultivation of microalgae and bacteria is a promising strategy for improving the overall efficiency of wastewater treatment and for achieving a complete removal of many types of toxic compounds. Nevertheless, the efficiency of the symbiotic system may be jeopardized when applied for the treatment of wastewater containing high concentrations of toxic pollutants. For instance, although complete degradation of phenol has been observed in a microalgae-activated sludge system, the concentration of phenol was normally below 600 ppm. For influents that contain a high level of phenol such as that produced in a coking plant, the active function of bacteria might be inhibited [144]. The mechanism of membrane fouling mitigation in the microalgae-bacteria consortium still needs further investigation. Furthermore, the control of the system stability is challenging due to the complication of the interaction between the microalgae and bacteria, which in turn affects the setup efficiency.

While several innovations have been reported, the approaches for fouling mitigation in MPBRs still have room for improvements. The introduction of an electric field in photobioreactors can suppress fouling, but the electric field applied raises the operating cost of the system. Furthermore, the effects induced by the external electric field on the nutrient removal pathway need to be verified through further electrochemical analyses of the biomass. It is also necessary to assess the specific energy consumption of the system. The combination of microalgae wastewater treatment with a UV-activated photocatalytic process has been reported in a recent study [145]. The glass-supported TiO_2_ was used as the post-treatment of the photobioreactor to achieve a better removal of COD and organic compounds in highly loaded winery wastewater. While such a post-treatment concept is beneficial for enhancing the overall wastewater treatment efficiency, the use of a photocatalytic membrane provides an opportunity to directly integrate a photocatalytic system into the photobioreactor, eliminating the need for an additional post-treatment unit. The development of a photocatalytic membrane, a new generation of mixed matrix membrane which combines membrane filtration and photocatalytic degradation in a single entity, is becoming a prevailing method in membrane research due to its great potential in wastewater treatment [146]. With the ability of the membrane-embedded photocatalysts to photodegrade organic pollutants and hence mitigate the fouling issue, the integration of such photocatalytic membranes is expected to expand the application of microalgae-based MPBRs for more challenging complex wastewater treatments. The exploration of photocatalytic membranes with solar light harvesting will be an important tool to promote the economic feasibility of the integrated system.

The successful implementation of an innovative technology is not only judged based on its ability to achieve the desired outcome to solve engineering issues but is also evaluated based on the cost effectiveness of the entire process. Furthermore, the wastewater pre-treatment costs involved must also be considered in the final economics. The economic feasibility of the entire MPBR system has rarely been reported in the literature, and studies focused on a detailed cost analysis of the entire wastewater treatment are still limited. In addition to the economic concern, the operation of MPBRs in outdoor conditions is also challenged by many technical issues. When operated outdoors, the efficiency of the system is highly susceptible to environmental factors such as the surrounding temperature and sunlight intensity. With the fluctuations in the environmental parameters, the nutrient uptake by microalgae is expected to reduce when moving from the bench-scale MPBR to an outdoor setting. It has been reported that the nutrient recovery efficiency and biomass productivity was reduced by a factor of 1–3 and 10–13, respectively, under outdoor conditions [147]. The current investigations on microalgae cultivation in MPBRs have not been focused on optimizing these external parameters. The system and set up of this application are in fact important for the assessment of the biomass yield and nutrient recovery efficiency of microalgae. Therefore, more studies are required to elucidate the operational issues so that the baselines for the future improvement of MPBRs can be established in a more relevant environment, with respect to wastewater treatment and nutrient recovery in microalgae. It is suggested that economic and environmental assessments be conducted in different outdoor settings that account for fluctuating environmental conditions that could affect biomass productivity and nutrient recovery efficiencies. As process optimization is certainly required to attain higher nutrient removal efficiencies and biomass productivities, the optimization performed under such realistic settings will be more representative for practical references.

Energy consumption is another issue associated with the sustainability of membrane processes for large-scale applications. Low-pressure membrane processes such as FO have been increasingly used as an alternative to pressure-driven processes to minimize the energy consumption and membrane fouling tendency. However, there are still doubts on the claims pertaining to the overall energy consumption of OMBRs. The mitigation of inherent issues of FO such as external concentration polarization and reverse solute migration are closely related to the draw solution and energy input of the process. Despite the attractive features demonstrated, FO can only outperform other pressure-driven counterparts if there is no extensive energy requirement for the draw solution recovery. Although systematic comparisons of the performances and energy efficiencies of conventional MBR and OMBR systems operated with microalgae for wastewater treatment have been accomplished, the challenges related to the draw solution recovery in microalgae osmotic bioreactor systems have not been practically addressed. The fouling formation and mechanisms become more complicated when cultivating microalgae in wastewater, as a huge variety of microorganisms are present in wastewater. The implementation of a co-cultivation of microalgae and bacteria is a double-edged sword; it may worsen the fouling of the membrane when excessive EPS and soluble microbial products are produced as a result of the environmental stress and competition between microalgae and bacteria. Therefore, the growth and ratio of microalgae and bacteria in the consortium should be carefully controlled. A better understanding of the effect of the microalgae–wastewater suspension on the membrane fouling and establishment of effective membrane fouling mitigation are of great importance.

Nutrient recovery using microalgae-based MPBRs should not be restricted to using wastewater as a nutrient source. Urine has also been identified as a potential nutrient source for microalgae cultivation due to its high load of nitrogen and phosphorus. Furthermore, although the wastewater treatment and nutrient recovery efficiency of MPBRs have been increasingly investigated, studies on algal lipid production in MPBRs are still scarce. It has been reported that the nutrient concentration in secondary effluents is too inconsistent to sufficiently support a satisfactory micro algal biomass production in a batch culture mode [148]. To realize the utilization of the microalgae biomass as a renewable feedstock, it is necessary to provide more insights into the microalgal lipid accumulation properties during MPBR operation so that the production capacity of algal lipids can be improved. In addition, such a system should not be limited to the recovery of water and nutrients; carbon dioxide and thermal energy can also be potentially recovered. A carbon dioxide-selective membrane allows for the enrichment of carbon dioxide gas which can be directly fed into the MPBR to sustain the growth of microalgae through photosynthesis.

## 8. Concluding Remarks

The implementation of innovative wastewater treatment systems is expected to take place in line with the adoption of a circular economy, which enforces stringent regulations for wastewater discharge. The increasing emphasis in water reclamation for reuse in many industries further promotes this development. As wastewater has been increasingly regarded as a significant source of nutrient, water, and energy, the existing industrial and municipal water treatment processes have shifted to a new paradigm where the target of wastewater treatment is no longer has sole emphasis on pollutant removal but also emphasizes adding value through nutrient recovery and energy production. This review discusses the recent development of microalgae-MPBR to simultaneously realize wastewater treatment, nutrient recovery, and biomass production in a single step. Emphases have been given on the innovations made in the design of photobioreactors and membranes and on the optimization of parameters to counter the existing issues that hamper its application. More laboratory trials and pilot-scale validations are required to fix the technological glitches so that the microalgae-based MPBR can be truly known as an economically and environmentally sustainable alternative for wastewater treatment. As a serious contender to conventional wastewater treatment approaches, this approach will serve as a sustainable and resilient component in water and wastewater treatment-related industries. Depending upon the pace of innovations in this area, it is expected that the technology will become more prevailing and mature in the next 5–10 years.

## Figures and Tables

**Table 1:** Summary of the nutrient removal efficiencies of MPBRs.

System	Membrane/Configuration	Microalgae	Wastewater	N Removal Efficiency/Rate	P Removal/Efficiency/Rate	Ref
MPBR	PVDF hollow fiber	*Chaetophora* sp., *Navicula* sp.	Synthetic secondary water	30.25%	40.58%	[104]
MPBR	Flat plate	*C. vulgaris*	Synthetic municipal wastewater	76.7%	66.2%	[105]
Pilot outdoor MPBR	Hollow fiber	*C. vulgaris*, *Scenedesmus* sp.	Anaerobic MBR effluent	7.68 mg/L.d	1.17 mg/L.d	[106]
Pilot outdoor MPBR	Hollow fiber	*Scenedesmus* sp.	Anaerobic MBR effluent	29.7 mg/L.d	3.8 mg/L.d	[108]
OMPBR	HTI TFC hollow fiber	*C. vulgaris*	Synthetic tertiary wastewater	93%	89%	[109]
OMPBR	Flat sheet TFC	*C. vulgaris*	Synthetic wastewater	100%	98.7%	[111]
MPBR	Ag/GO PVDF membrane	*C. vulgaris*	Synthetic municipal wastewater	92.3%	66.1%	[132]
MPBR	Cellulose ester hollow fiber	*C. vulgaris-activated sludge*	Raw treatment plant wastewater	94.36%	88.37	[128]
MPBR	PVDF flat sheet	*C. vulgaris-activated sludge*	Synthetic domestic wastewater	92.7%	92.4%	[126]
Electrokinetic-assisted MPBR	PVDF flat sheet	*C. vulgaris*	Synthetic municipal wastewater	41.81%	97.98%	[138]
Electrokinetic-assisted MPBR	PVDF hollow fiber	*C. vulgaris-activated sludge*	Synthetic municipal wastewater	>98% *	>98% *	[139]
Annular two-chamber MPBR	Ion exchange membranes	*C. vulgaris*	Dairy manure wastewater	8.95 mg/L.d	2.31 mg/L.d	[140]

* Value estimated from graphs.

## Data Availability

Not applicable.

## List of Abbreviations

AOM	Algal organic matters
COD	Chemical oxygen demand
EOM	External organic matters
EPS	Extracellular polymeric substances
FO	Forward osmosis
HRT	Hydraulic retention time
IOM	Internal organic matters
MPBR	Membrane photobioreactor
OMBR	Osmotic membrane bioreactor
OMPBR	Osmotic membrane photobioreactor
PBR	Photobioreactors
PVDF	Polyvinylidene fluoride
RO	Reverse osmosis
Ag/GO	Silver/graphene oxide
SRT	Solids retention time
TiO_2_	Titanium dioxide
TMP	Transmembrane pressure
TEP	Transparent exopolymer particles
